# Tunable Multi-Functional Metamaterial Based on Photosensitive Silicon for Unidirectional Reflectionlessness, Polarization Conversion, and Asymmetric Transmission

**DOI:** 10.3390/ma18112614

**Published:** 2025-06-03

**Authors:** Xue Ren, Yiwen Zhang, Yingqiao Zhang, Xingri Jin

**Affiliations:** Department of Physics, College of Science, Yanbian University, Yanji 133002, China

**Keywords:** unidirectional reflectionlessness, polarization conversion, asymmetric transmission, metamaterial

## Abstract

We propose a tunable multi-functional metamaterial composed of two pairs of gold corner resonators interconnected with photosensitive silicon, operating in the terahertz range. This design achieves dual-band unidirectional reflectionlessness, polarization conversion, and asymmetric transmission for linearly polarized waves, regardless of whether the photosensitive silicon is in the insulating or conductivity state. When the photosensitive silicon transitions from the insulating state to the conductivity state, its conductivity increases significantly, resulting in a frequency shift phenomenon in the functional peak frequencies. Numerical simulations demonstrate the structure’s robust performance in dual-band unidirectional reflectionlessness and its significant asymmetric transmission, with minimal sensitivity to variations in the incident angle and photosensitive silicon sheet length. By integrating multiple functionalities and enabling frequency tunability through the control of photosensitive silicon conductivity, this design offers a reconfigurable solution for THz applications, such as switches, polarization converters, and modulators.

## 1. Introduction

Terahertz (THz) waves, spanning a frequency range of 0.1 THz to 10 THz, occupy the transitional region of macroscopic electronics and microscopic photonics within the electromagnetic spectrum, which forms the boundary between microwaves and infrared radiation [1,2]. THz waves exhibit exceptional properties, including a broad bandwidth, high penetration efficiency, low photon energy, and strong coherence [3,4,5,6]. However, the lack of functional materials that can efficiently interact with THz waves has severely limited their practical applications, as most natural materials show only weak interactions in this frequency range. In this context, metamaterials have emerged as a promising solution. They are artificially engineered periodic structures made of subwavelength unit cells, enabling unique control over THz waves [7,8,9]. At specific THz frequencies, metamaterials exhibit unique physical properties that are unattainable with traditional materials, such as negative refraction [10], electromagnetic invisibility [11,12,13], and electromagnetic shielding [14]. As research in this field progresses, precise control over the amplitude, phase, and polarization of electromagnetic waves has become achievable through the meticulous design of the shape, size, and arrangement of the unit cells within metamaterials [15,16]. Moreover, metamaterials have been instrumental in the design and fabrication of optical devices with advanced functionalities, including unidirectional reflectionlessness (UR) [17,18,19], polarization conversion (PC) [20], and asymmetric transmission (AT) [21,22,23]. For instance, a metamaterial composed of double-layer double i-shaped resonators has been proposed, enabling control over UR and AT by adjusting the angle of incidence [24]. In the THz regime, a broadband bi-functional metasurface combining a double-split-ring resonator with a bar resonator has been designed to significantly enhance the bandwidth and efficiency of PC and AT [25]. Additionally, an ultra-broadband multilayer metasurface, resembling a Fabry–Perot cavity, has demonstrated ultra-broadband cross-polarization conversion and efficient AT, with both functionalities remaining stable at an incidence angle of 0∼45° [26]. However, once the geometric parameters of these structures are fixed, their performance does not change. To overcome this limitation, researchers have integrated tunable materials into metamaterials, thereby combining tunability with multifunctionality [27].

For tunable THz metamaterials, the key to achieving tunability lies in utilizing active materials to modulate conductivity [28]. So far, three typical active materials have emerged for tunable THz metamaterials: vanadium dioxide (VO_2_) [29,30,31,32], graphene [33,34,35], and photosensitive silicon (PSi) [36,37,38]. PSi enables fast optical tuning without thermal constraints, unlike VO_2_, and it offers easier fabrication when compared to graphene’s complex doping requirements [39,40]. In contrast, PSi has been widely recognized due to its conductivity modulation mechanism, where the conductivity can be tuned by adjusting the intensity of the pump beam [41]. A tunable metamaterial was proposed based on silicon state modulation [42]. In the insulating state, the metamaterial achieved a polarization conversion ratio (PCR) exceeding 0.9 within 3.82 THz to 4.43 THz, while in the conductivity state, it exhibited broadband absorption from 1.45 THz to 3.36 THz. Additionally, a dual-frequency switch utilizing PSi was developed [39], where the conductivity was modulated by varying the pump beam intensity to enable switching between on and off states. Up to now, although numerous designs of multifunctional metamaterials have been proposed, achieving metamaterials that simultaneously possess tunability, UR, PC, and AT optical properties remains a significant challenge.

In this study, we propose a tunable multi-functional THz metamaterial based on PSi that integrates UR, PC, and AT for linearly polarized waves. When PSi acts as an insulator with a conductivity of 1 S/m, the structure demonstrates dual-band UR and PC capabilities, exhibiting higher PCRs for both *x*- and *y*-polarized waves. Conversely, when PSi is in the conductivity state with a conductivity of 1 × 10^5^ S/m, the metamaterial achieves all desired functionalities across a broader frequency range, accompanied by frequency shifts in UR, PC, and AT. Additionally, better AT parameters are achieved for both *x*- and *y*-polarized waves. This highlights the versatility, superior performance, and adjustability of the design.

## 2. Structure Design and Methods

**Figure 1:**
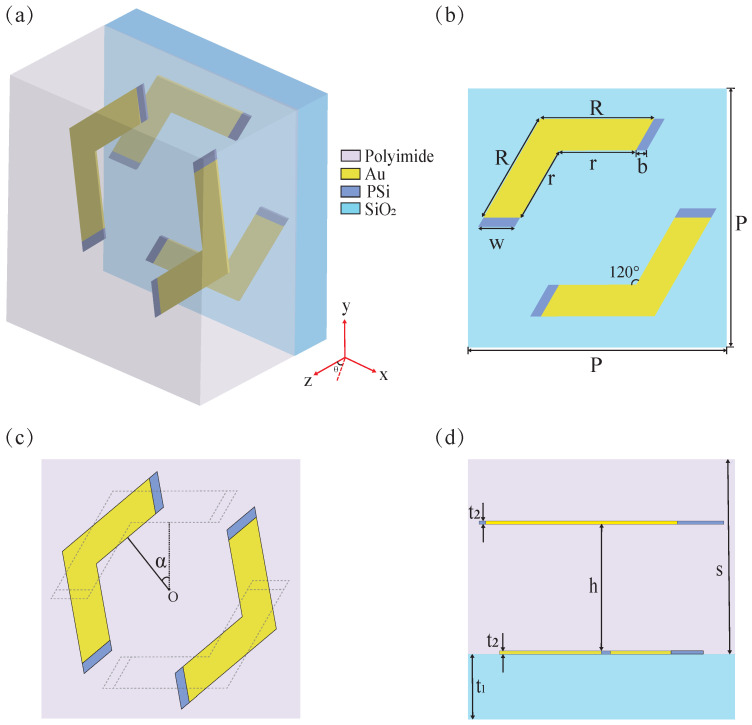
(**a**) Unit cell diagram of metamaterial. (**b**) Front view of the lower resonator. (**c**) Front view of the upper resonator. (**d**) Side view of the unit cell in the *y*-*z* plane.

Figure 1 illustrates the geometric configuration and dimensional specifications of the proposed metamaterial unit cell, featuring a composite structure of PSi integrated with two pairs of gold corner resonators. Figure 1a depicts the unit cell configuration, which comprises a SiO_2_ substrate with a dielectric constant of 2.07 [43]. The polyimide dielectric layer, characterized by a dielectric constant of 2.4 and a loss tangent of 0.005, encapsulates two pairs of gold corner resonators integrated with PSi. Figure 1b,c show front views of the lower and upper resonators, respectively. Figure 1b shows the unit cell structure of the metamaterial. Here, the values of the geometric parameters are defined as follows: P = 100 μm, R = 44 μm, r = 30 μm, w = 14 μm, and b = 4 μm. The two pairs of metallic corner resonators are geometrically identical and symmetrical about the central point O. The upper resonator is generated by rotating the lower one counterclockwise by an angle of α = 40°, as illustrated in Figure 1c. Figure 1d presents a side view of the unit cell in the *y*-*z* plane, illustrating the layered structure with precise dimensional parameters. The substrate thickness (t_1_) measures 10 μm, while the resonator and dielectric layer have thicknesses of t_2_ = 0.2 μm and s = 30 μm, respectively. Additionally, the vertical separation distance (h) between the upper and lower resonators is maintained at 20 μm. Numerical simulations are conducted using CST Microwave Studio, employing the frequency-domain finite integration technique. The boundary conditions are configured with unit cell periodicity along both the *x*- and *y*-axes, while an open boundary condition is applied on the *z*-axis. In the simulation, the conductivity of gold is set to 4.561 × 10^7^ S/m, while the dielectric constant of PSi is assigned a value of 11.9 [44]. Moreover, its conductivity demonstrates a dynamic dependence on the power of the applied optical pump beam. Under illumination by a near-infrared laser pulse centered at 800 nm, which serves as the optical pump for carrier generation in PSi, the conductivity of PSi varies with the incident energy flux. This relationship can be mathematically expressed as follows: $\sigma_{\mathrm{PSi}}$ = 4.863 × 10^−4^ × I^2^ + 0.1856 × I + 1.569 [45,46], where $\sigma_{\mathrm{PSi}}$ denotes the conductivity of PSi, and I represents the pump beam power. In the absence of optical pumping (I = 0), the conductivity of PSi measures at approximately 1 S/m. However, when subjected to a pump beam power of 294.6 μJ/cm^2^, the conductivity undergoes a significant enhancement, reaching approximately 1 × 10^5^ S/m. A time delay of near 5 ps between optical pumping and the terahertz probe beam is defined experimentally. This delay ensures a quasi-steady state for the charge carriers of Si because the lifetime of a few hundreds of nanoseconds is orders of magnitude longer than the picosecond duration of the THz pulse [46,47,48].

To facilitate an analysis of the reflection and transmission characteristics of the electromagnetic waves in the structure, we use the Jones matrix to establish the relationships between the incident field and the reflected field, as well as the transmitted field [49].

(1) $$E_{i}\left(r,t\right)=\left(\begin{array}{c} I_{x}\\ I_{y} \end{array}\right)e^{i(kz-wt)},$$

(2) $$E_{r}\left(r,t\right)=\left(\begin{array}{c} r_{x}\\ r_{y} \end{array}\right)e^{i(kz-wt)},$$

(3) $$E_{t}\left(r,t\right)=\left(\begin{array}{c} t_{x}\\ t_{y} \end{array}\right)e^{i(kz-wt)},$$

where ω represents the angular frequency of the incident wave, and *k* denotes the wavenumber. The complex amplitudes of the incident wave along the *x*- and *y*-directions are given by $I_{x}$ and $I_{y}$, respectively. Correspondingly, $r_{x}$ and $r_{y}$ signify the complex amplitudes of the reflected wave in the *x*- and *y*-directions, while $t_{x}$ and $t_{y}$ represent those of the transmitted wave, respectively. Additionally, the Jones matrices utilized to characterize the relationships between the transmitted wave, the reflected wave, and the incident linearly polarized wave can be expressed as

(4) $$\left(\begin{array}{c} t_{x}\\ t_{y} \end{array}\right)=\left(\begin{array}{cc} t_{xx} & t_{xy}\\ t_{yx} & t_{yy} \end{array}\right)\left(\begin{array}{c} I_{x}\\ I_{y} \end{array}\right)=T_{lin}^{f}\left(\begin{array}{c} I_{x}\\ I_{y} \end{array}\right),$$

(5) $$\left(\begin{array}{c} r_{x}\\ r_{y} \end{array}\right)=\left(\begin{array}{cc} r_{xx} & r_{xy}\\ r_{yx} & r_{yy} \end{array}\right)\left(\begin{array}{c} I_{x}\\ I_{y} \end{array}\right)=R_{lin}^{f}\left(\begin{array}{c} I_{x}\\ I_{y} \end{array}\right),$$

where the subscript “lin” refers to the linearly polarized wave, and the superscript “*f*” indicates the forward (−z) incidence. The terms $t_{xx}$ ($t_{yy}$) and $r_{xx}$ ($r_{yy}$) represent the transmitted and reflected waves in the *x* (*y*)-direction when the polarization of the incident wave is along the *x* (*y*)-direction. Similarly, $t_{xy}$ ($t_{yx}$) and $r_{xy}$ ($r_{yx}$) denote the transmitted and reflected waves in the *x* (*y*)-direction when the polarization of the incident wave is along the *y* (*x*)-direction. Based on the reciprocity theorem, the Jones matrices for backward (+z) propagation can be expressed as follows:

(6) $$T_{lin}^{b}=\left(\begin{array}{cc} t_{xx} & -t_{yx}\\ -t_{xy} & t_{yy} \end{array}\right),$$

(7) $$R_{lin}^{b}=\left(\begin{array}{cc} r_{xx} & -r_{yx}\\ -r_{xy} & r_{yy} \end{array}\right).$$

So, the total transmissions and reflections of the *x*- and *y*-polarized incident waves along the forward and backward directions can be written, respectively, as

(8) $$T_{x(y)}^{f(b)}={\left|t_{xx(yy)}^{f(b)}\right|}^{2}+{\left|t_{yx(xy)}^{f(b)}\right|}^{2},$$

(9) $$R_{x(y)}^{f(b)}={\left|r_{xx(yy)}^{f(b)}\right|}^{2}+{\left|r_{yx(xy)}^{f(b)}\right|}^{2}.$$

In addition, the performance of metamaterial in PC is characterized by the PCR, defined as follows [50]:

(10) $${\mathrm{PCR}}^{f}=\frac{|t_{yx}{|}^{2}}{|t_{yx}{|}^{2}+|t_{xx}{|}^{2}},$$

(11) $${\mathrm{PCR}}^{b}=\frac{|t_{xy}{|}^{2}}{|t_{xy}{|}^{2}+|t_{yy}{|}^{2}}.$$

Additionally, the AT effect of the metamaterial can be characterized by the AT parameter △. This parameter quantifies the difference in the total transmissions for the same polarized wave between the forward and backward directions, and that for polarized waves can be derived directly from the Jones matrix as follows:

(12) $$\Delta_{lin}^{x}=T_{x}^{f}-T_{x}^{b}=|t_{xx}^{f}{|}^{2}+|t_{yx}^{f}{|}^{2}-|t_{xx}^{b}{|}^{2}-{\left|t_{yx}^{b}\right|}^{2},$$

(13) $$\Delta_{lin}^{y}=T_{y}^{f}-T_{y}^{b}=|t_{yy}^{f}{|}^{2}+|t_{xy}^{f}{|}^{2}-|t_{yy}^{b}{|}^{2}-{\left|t_{xy}^{b}\right|}^{2}=-{▵}_{lin}^{x}.$$

## 3. Results and Discussion

### 3.1. Unidirectional Reflectionlessness

**Figure 2:**
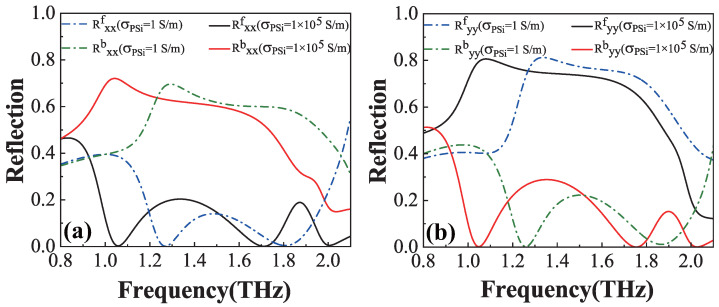
The co-polarized reflections for the *x*-polarized wave (**a**) and the *y*-polarized wave (**b**) for the forward and backward directions, respectively, when PSi is in the insulating state or conductivity state.

Figure 2a,b show the co-polarized reflection spectra for the forward and backward directions under the *x*- and *y*-polarized waves, respectively. In these figures, the dash–dotted and solid lines correspond to the cases of the insulating ($\sigma_{\mathrm{PSi}}$ = 1 S/m) and conductivity ($\sigma_{\mathrm{PSi}}$ = 1 × 10^5^ S/m) states of PSi, with the following color-coded representations: blue (black) and green (red) dash–dotted (solid) lines indicate forward and backward reflections in the insulating (conductivity) state of PSi, respectively, for *x*- and *y*-polarized waves. For the *x*-polarized wave (Figure 2a), the insulating state of PSi demonstrates co-polarized reflection $R_{xx}^{b}$ values of approximately 0.69 and 0.59 at 1.27 THz and 1.79 THz (green dash–dotted line), where $R_{xx}^{f}$ remains near zero (blue dash–dotted line), respectively. In the conductivity state of PSi, the $R_{xx}^{b}$ value reaches about 0.71 and 0.51 at 1.06 THz and 1.71 THz (red solid line), with $R_{xx}^{f}$ maintaining a near-zero value (black solid line), clearly indicating the dual-band UR phenomenon for the *x*-polarized wave. Regarding the *y*-polarized wave (Figure 2b), the insulating state of PSi shows co-polarized reflection $R_{yy}^{f}$ values of approximately 0.75 and 0.61 at 1.26 THz and 1.86 THz (blue dash–dotted line), where $R_{yy}^{b}$ approaches zero (green dash–dotted line), respectively. In the conductivity state of PSi, the $R_{yy}^{f}$ value increases to about 0.80 and 0.65 at 1.05 THz and 1.76 THz (black solid line), with $R_{yy}^{b}$ remaining nearly zero (red solid line), confirming the dual-band UR phenomenon for the *y*-polarized wave. These observations demonstrate that the UR phenomenon can be effectively modulated through PSi state transitions, exhibiting distinct red-shift behavior when PSi changes from the insulating to the conductivity state. Therefore, the structure exhibits robust dual-band UR characteristics.

**Figure 3:**
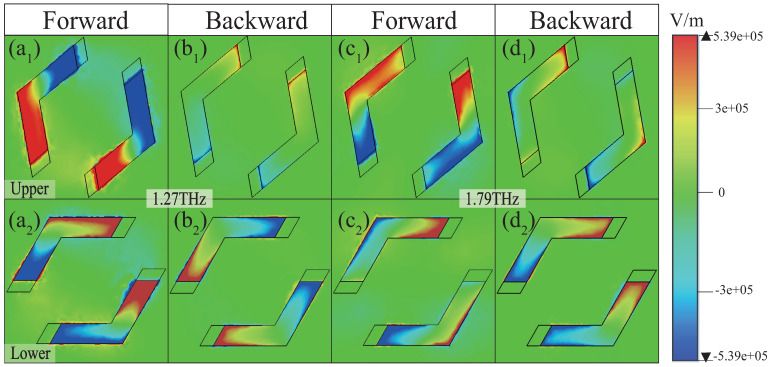
The *z*-component electric field distributions for the upper (**a**_1_–**d**_1_) and lower (**a**_2_–**d**_2_) resonators at 1.27 THz and 1.79 THz for the *x*-polarized wave along the forward and backward directions, respectively.

**Figure 4:**
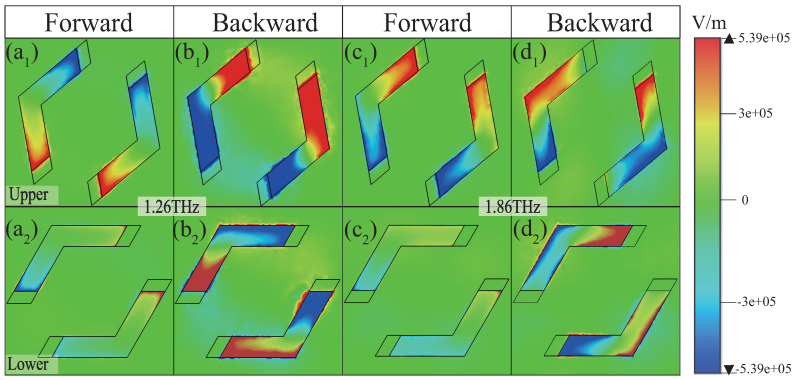
The *z*-component electric field distributions for the upper (**a**_1_–**d**_1_) and lower (**a**_2_–**d**_2_) resonators at 1.26 THz and 1.86 THz for the *y*-polarized wave along the forward and backward directions, respectively.

To gain deeper insights into the generation mechanism of UR, the *z*-component electric field distributions are presented in Figure 3 and Figure 4 for PSi in the insulating state. For conciseness, the analysis focuses solely on the electric field distributions of the *x*- and *y*-polarized waves in the insulating state of PSi. Figure 3 illustrates the electric field distributions of the upper and lower resonators for the *x*-polarized wave at frequencies of 1.27 THz and 1.79 THz, respectively. As shown in Figure 3(a_1_,a_2_) at 1.27 THz, when the *x*-polarized wave is incident in the forward direction, both the upper and lower resonators are strongly excited with a phase difference approaching π, leading to near-zero reflection (Figure 2a, blue dash–dotted line) due to destructive interference. Conversely, Figure 3(b_1_,b_2_) reveal that, for backward incidence, the upper resonator is weakly excited, while the lower resonator is strongly excited, resulting in a high reflection of ∼0.69 (Figure 2a, green dash–dotted line). A similar behavior is observed at 1.79 THz, as depicted in Figure 3(c_1_–d_2_), confirming the UR phenomenon at this frequency. In contrast, for the *y*-polarized wave, the excitation characteristics differ significantly. As demonstrated in Figure 4(a_1_–d_2_), in the forward direction, the upper resonator is strongly excited, while the lower resonator is weakly excited, yielding a high reflection of ∼0.75 and ∼0.61 at 1.26 THz and 1.86 THz, respectively (Figure 2b, blue dash–dotted line). However, in the backward direction, both resonators are strongly excited, with the phase difference close to π, resulting in near-zero reflection at these frequencies (Figure 2b, green dash–dotted line). Through a detailed analysis of the electric field distributions, the dual-band UR phenomenon is clearly observed, highlighting the critical role of resonator excitations and phase difference in achieving UR.

### 3.2. Polarization Conversion

**Figure 5:**
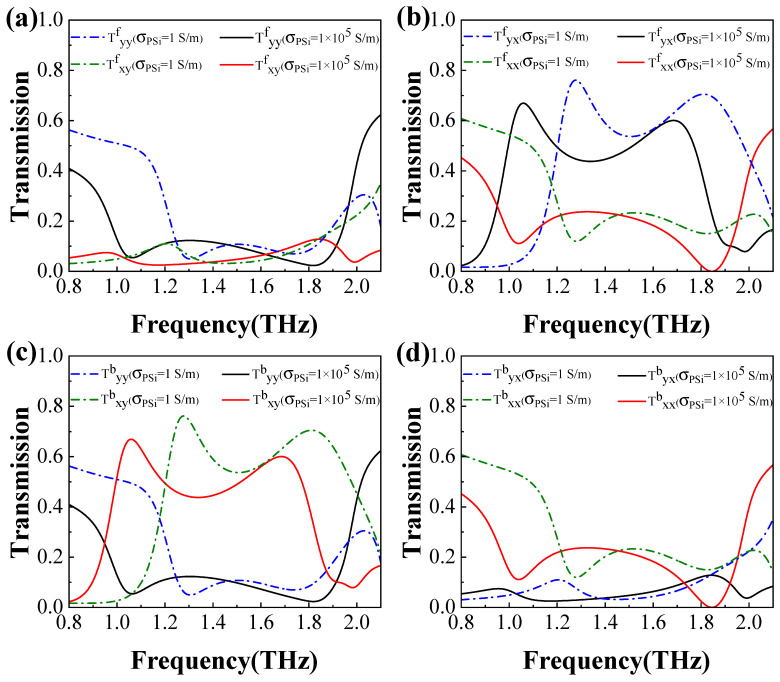
The transmission characteristics of linearly polarized waves for both the insulating and conductivity states of PSi. The transmission spectra of the *y*-polarized wave incident in the forward (**a**) and backward (**c**) directions, as well as the *x*-polarized wave incident in the forward (**b**) and backward (**d**) directions.

Next, we analyze the PC characteristics of the transmission waves in both the forward and backward directions for the *x*- and *y*-polarized incident waves, as illustrated in Figure 5. In Figure 5a,c, it is observed that, when PSi is in the insulating state, the cross-polarized transmission $T_{xy}^{b}$ exceeds 0.6 within the frequency ranges of 1.22 ∼1.39 THz and 1.64∼1.90 THz, while in the corresponding frequency ranges, the transmissions $T_{yy}^{f}$, $T_{xy}^{f}$, and $T_{yy}^{b}$ remain below 0.20. In contrast, when PSi is in the conductivity state, $T_{xy}^{b}$ surpasses 0.6 in the frequency ranges of 1.02∼1.12 THz and 1.67∼1.70 THz, while in the corresponding frequency ranges, the transmissions $T_{yy}^{f}$, $T_{xy}^{f}$, and $T_{yy}^{b}$ remain below 0.20. This demonstrates that a *y*-polarized wave incident in the backward direction is predominantly converted into an *x*-polarized wave. Similarly, from Figure 5b,d, it is evident that the cross-polarized transmission $T_{yx}^{f}$ exceeds 0.6 in the frequency ranges of 1.22∼1.39 THz and 1.66∼1.92 THz for the forward direction when PSi is in the insulating state, while in the corresponding frequency ranges, the transmissions $T_{yx}^{b}$, $T_{xx}^{b}$, and $T_{xx}^{f}$ all remain below 0.2. When PSi is in the conductivity state, $T_{yx}^{f}$ exceeds 0.6 in the frequency ranges of 1.02∼1.12 THz and 1.67∼1.70 THz, while in the corresponding frequency ranges, the transmissions $T_{yx}^{b}$, $T_{xx}^{b}$, and $T_{xx}^{f}$ all remain below 0.2. This indicates that an *x*-polarized wave incident in the forward direction is predominantly converted into a *y*-polarized wave. These results highlight that the proposed structure not only achieves efficient PC but also exhibits exceptional AT performance.

**Figure 6:**
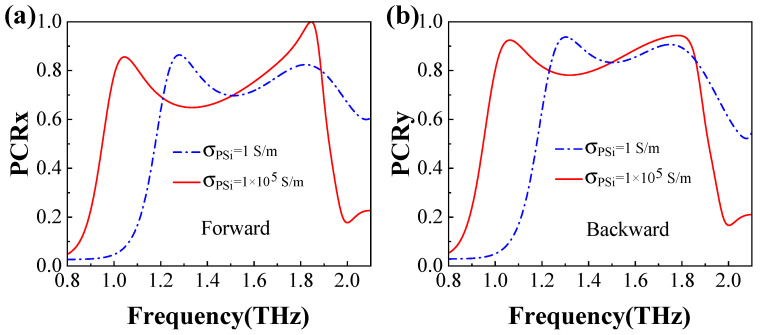
PCR_*x*_ for the forward direction (**a**) and PCR_*y*_ for the backward (**b**) direction, with PSi in the insulating and conductivity states, respectively.

The PCR is a critical parameter for evaluating the PC capability, as Equations (10) and (11) define. Transmission PCR curves versus frequency for both the insulating and conductivity states of PSi are illustrated in Figure 6a,b for the forward and backward directions, respectively, when the *x*- and *y*-polarized waves are incident. As shown in Figure 6a, when the *x*-polarized wave propagates in the forward direction with PSi in the insulating state, the PCR_*x*_ value exceeds 0.8 within the frequency ranges of 1.23∼1.35 THz and 1.74∼1.88 THz. The peak values of PCR_*x*_ reach approximately 0.86 and 0.82 at 1.28 THz and 1.82 THz, respectively. When PSi is in the conductivity state, the PCR_*x*_ values for the frequency ranges of 1.00∼1.10 THz and 1.66∼1.88 THz also surpass 0.8, with peak values of approximately 0.86 and 0.99 at 1.04 THz and 1.84 THz, respectively. Similarly, for the *y*-polarized wave incident in the backward direction with PSi in the insulating state (Figure 6b), the PCR_*y*_ values exceed 0.9 within the frequency ranges of 1.26∼1.36 THz and 1.70∼1.78 THz. The peak PCR_*y*_ values reach approximately 0.94 and 0.90 at 1.30 THz and 1.74 THz, respectively. When PSi is in the conductivity state, the PCR_*y*_ values for the frequency ranges of 1.03∼1.10 THz and 1.63∼1.84 THz also exceed 0.9, with peak values of approximately 0.93 and 0.94 at both 1.06 THz and 1.78 THz, respectively. These results clearly demonstrate that, when the *x* (*y*)-polarized wave propagates in the forward (backward) direction, the majority of the *x* (*y*)-polarized wave is converted into a *y* (*x*)-polarized wave. These findings confirm that the designed metamaterial exhibits a good PC capability.

### 3.3. Asymmetric Transmission

**Figure 7:**
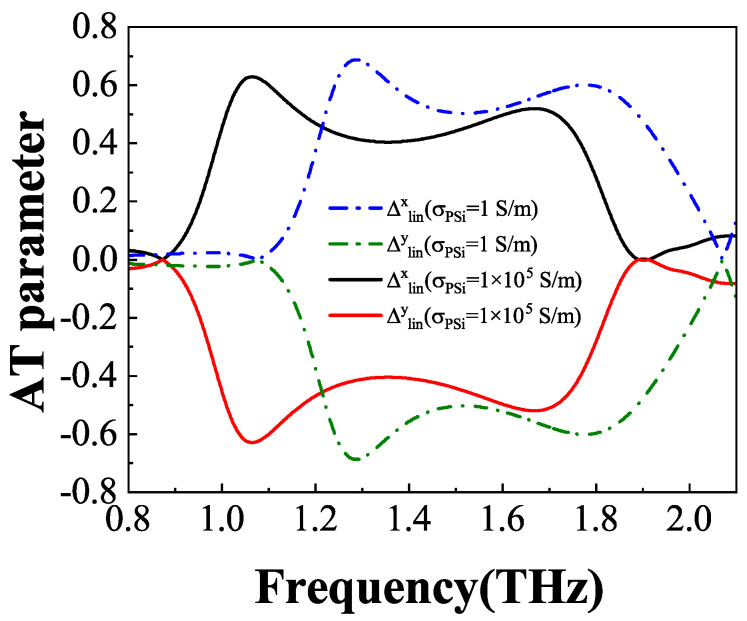
The AT parameters of the *x*- and *y*-polarized incident waves under the insulating and conductivity states of PSi.

Curves of the AT parameter, denoted as Δ, are plotted in Figure 7 based on Equations (12) and (13) for both the insulating and conductivity states of PSi. Notably, the AT parameters Δ display entirely opposite trends for the *x*- and *y*-polarized waves. Specifically, when PSi is in the insulating (conductivity) state, the values of $\Delta_{lin}^{x}$ are approximately 0.69 (0.63) and 0.60 (0.52) at frequencies of 1.29 THz (1.06 THz) and 1.77 THz (1.67THz), respectively, while the corresponding values of $\Delta_{lin}^{y}$ are approximately −0.69 (−0.63) and −0.60 (−0.52). These findings demonstrate that the AT effect can be effectively achieved for both *x*- and *y*-polarized waves, underscoring the polarization-independent AT characteristics of the structure.

### 3.4. The Effect of Incident Angle and PSi Sheet Length on UR and AT

**Figure 8:**
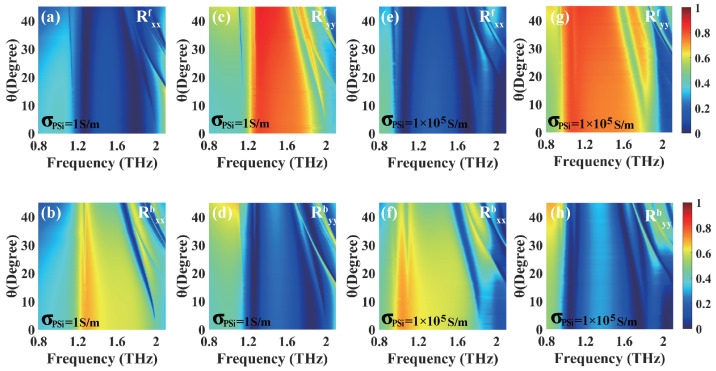
The dependences of the co-polarized reflections of the linearly polarized incident waves on the incident angle θ and the frequency for both the forward and backward directions when PSi is in the insulating state (**a**–**d**) and conductivity state (**e**–**h**), respectively, in the case of PSi sheet length b = 4 μm.

To investigate the influences of the incident angle θ and the length b of the PSi sheet on UR, we plot the co-polarized reflections of the linearly polarized incident waves as the functions of the incident angle θ and the length b of the PSi sheet, respectively. Figure 8a–d illustrate the co-polarized reflections for the forward and backward directions of the *x*- and *y*-polarized waves versus the incident angle θ and frequency when PSi is in the insulating state. As shown in Figure 8a,d, the co-polarized reflection $R_{xx}^{f}$($R_{yy}^{b}$) approaches nearly zero around 1.27 THz (1.26 THz) and 1.79 THz (1.86 THz) within the incident angle range of 0∼$45^{{}^{\circ}}$, which corresponds to a high reflection $R_{xx}^{b}$($R_{yy}^{f}$) in Figure 8b,c. From Figure 8a–d, it is evident that dual-band UR phenomena occur for both the *x*- and *y*-polarized waves. Similarly, when PSi is in the conductivity state, as shown in Figure 8e,f, the co-polarized reflection $R_{xx}^{f}$ approaches nearly zero around frequencies of 1.06 THz and 1.71 THz within the incident angle range of ${0∼45}^{{}^{\circ}}$, while $R_{xx}^{b}$ exhibits a relatively high reflection. In Figure 8g,h, around frequencies of 1.05 THz and 1.76 THz, the co-polarized reflection $R_{yy}^{b}$ approaches nearly zero within the incident angle range of 0∼$45^{{}^{\circ}}$, while $R_{yy}^{f}$ exhibits a relatively high reflection. These results demonstrate that the proposed structure exhibits dual-band UR phenomena over wide ranges of the incident angle.

**Figure 9:**
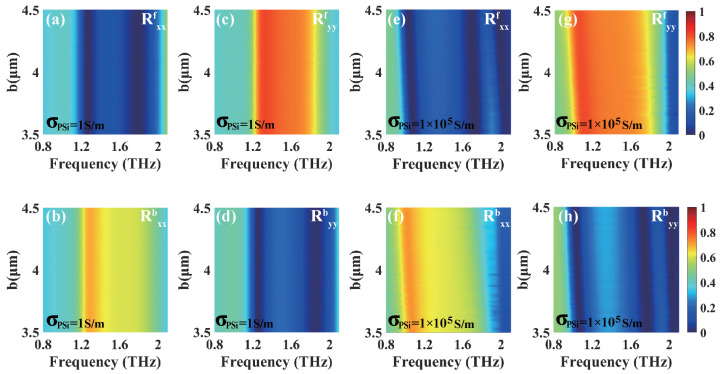
The dependences of the co-polarized reflections on the length b of the PSi sheet and frequency for both the forward and backward directions when PSi is in the insulating state (**a**–**d**) and conductivity state (**e**–**h**), respectively, in the case of an incident angle of θ = 0°.

The co-polarized reflection spectra of the *x*- and *y*-polarized waves for both the forward and backward directions are presented in Figure 9, respectively, showing the dependences on the length b of the PSi sheet for both the insulating and conductivity states. As depicted in Figure 9a–d, when PSi is in the insulating state, the co-polarized reflection $R_{xx}^{b}$ ($R_{yy}^{f}$) remains consistently high and stable at frequencies of 1.27 THz (1.26 THz) and 1.79 THz (1.86 THz) as the length b of the PSi sheet increases from 3.5 μm to 4.5 μm. In contrast, the co-polarized reflection $R_{xx}^{f}$ ($R_{yy}^{b}$) approaches nearly zero at the same frequencies. Similarly, when PSi is in the conductivity state, as illustrated in Figure 9e–h, the co-polarized reflection $R_{xx}^{b}$ ($R_{yy}^{f}$) demonstrates high stability at frequencies of 1.06 THz (1.05 THz) and 1.71 THz (1.76 THz) when increasing the length b of the PSi sheet from 3.5 μm to 4.5 μm, while $R_{xx}^{f}$ ($R_{yy}^{b}$) approaches nearly zero at the same frequencies. From Figure 9a–h, it is concluded that the structure exhibits dual-band UR over a wide range of PSi sheet lengths, and the peak positions of UR exhibit a slight red-shift.

**Figure 10:**
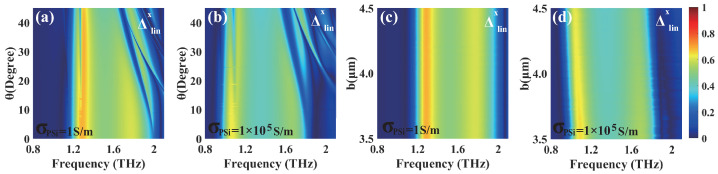
The AT parameter $\Delta_{lin}^{x}$ varied with the incident angle θ (**a**,**b**) and the length b of the PSi sheet (**c**,**d**) in the insulating (**a**,**c**) and conductivity (**b**,**d**) states of PSi when the length was b = 4 μm and the incident angle was θ = 0°, respectively.

Finally, we investigate the AT parameter by varying the incidence angle θ and the length b of the PSi sheet, with Figure 10 presenting a comprehensive analysis of its variations under different conditions. In the insulating state of PSi, the AT parameter demonstrates distinct characteristics across different frequencies. As shown in Figure 10a, the AT parameter maintains stable values of ∼0.69 and ∼0.60 around the frequencies of 1.29 THz and 1.77 THz when the incident angle θ is in the ranges of 0∼45° and 0∼26°, respectively, with b fixed at 4 μm. Furthermore, Figure 10c reveals that, when the PSi sheet length b is varied from 3.5∼4.5 μm, the AT parameter exhibits remarkable stability at values of ∼0.69 and ∼0.60 around the frequencies of 1.29 THz and 1.77 THz, respectively, with an incidence angle of θ = 0°. When PSi is in the conductivity state, the AT parameter demonstrates significant changes, maintaining stable values of ∼0.63 at 1.06 THz and ∼0.52 at 1.67 THz as the incident angle θ increases from 0∼45° and 0∼19°, with b fixed at 4 μm (Figure 10b), respectively. When the PSi sheet length b varies from 3.5∼4.5 μm at θ = 0° (Figure 10d), the AT parameter remains unchanged at ∼0.63 and ∼0.52 around 1.06 THz and 1.67 THz. Obviously, the AT parameter in the insulating state consistently exhibits higher values than that in the conductivity state, and the transition to the conductivity state induces red-shifts with peak values of the AT parameter. Notably, the AT parameter demonstrates remarkable stability to variations over wide ranges of the incident angle and PSi sheet length.

### 3.5. Relative Advantages and Potential Fabrication Processes

Compared with the existing literature summarized in Table 1 [51,52,53,54,55,56], the proposed structure exhibits significant advantages: it simultaneously achieves UR, PC, and AT while supporting dynamic tuning and enabling red-shifts of their peak frequencies. Moreover, the structure demonstrates robust performance against variations in wider ranges of the incident angle and PSi sheet length. These findings provide new insights for the design of multifunctional optical devices, highlighting their broad technological potential.

Next, we briefly discuss the feasibility of the fabrication process of the metamaterial structure. Firstly, gold films were deposited by electron beam evaporation on SiO_2_ substrate. After spin-coating photoresist, Au corner resonators were fabricated by patterning the Au film through photolithography with a photomask, followed by etching, and the residual photoresist was removed by plasma cleaning. Then, the PSi pattern was defined by secondary photolithography. After depositing the PSi film, it was lifted off to form the PSi structure connecting the Au corner, and then the dielectric layer was deposited to complete the lower resonator. Finally, the same steps were repeated to fabricate the upper resonator. As supported by a previous study [47,48], the proposed structure is feasible to fabricate under the current technical conditions.

## 4. Conclusions

We design a tunable THz multifunctional metamaterial using metal corner resonators connected with PSi, which integrates three key functionalities—unidirectional reflectionlessness, polarization conversion, and asymmetric transmission—in one. Remarkably, regardless of whether the PSi is in the insulating or conductivity state, the structure supports dual-band unidirectional reflectionlessness and polarization conversion for *x*- (*y*-) polarized waves, reaching a polarization conversion ratio PCR_*x*_ (PCR_*y*_) of up to 0.86 (0.94) in the the insulating state and 0.99 (0.93) in the conductivity state while maintaining higher AT parameters. The phase transition of PSi from the insulating to the conductivity state enables the tunability of the peak frequencies, accompanied by a slight red-shift. Furthermore, the structure exhibits robust unidirectional reflectionlessness and asymmetric transmission performances over broad incident angles (0∼45°) and PSi sheet lengths (3.5∼4.5 μm), demonstrating its exceptional performance stability and structural robustness. To summarize, this design overcomes the single-function constraint of traditional metamaterials by integrating unidirectional reflectionlessness, polarization conversion, and asymmetric transmission into a unified platform, thereby enabling the synergistic control of these functionalities within a single structure. Additionally, the functional peak frequency red-shift through the control of the conductivity state of PSi opens up possibilities for reconfigurable terahertz switches, polarization converters, modulators, and so on. While the numerical simulations in this study demonstrate the feasibility of our approach, we emphasize that experimental validation remains essential to fully verify these theoretical predictions and evaluate practical implementation challenges.

## Figures and Tables

**Table 1:** Comparison with other functional devices.

References	Incident Light Type	Tunable Material	Function	PC Type	Spectrum Range	Frequency Shift
[51]	LPL	PSi, VO_2_	PC, LTCPC	R, T	THz	No
[52]	LPL	PSi, graphene	T, A, R	No	THz	No
[53]	LPL	PSi, VO_2_	PC, A	T	THz	No
[54]	CPL	PSi	CDR, CDT	No	THz	No
[55]	LPL	PSi	EIT	No	THz	No
[56]	LPL	PSi	A	No	THz	Red-shift, blue-shift
This work	LPL	PSi	UR, PC, AT	T	THz	Red-shift

Explanation of abbreviations: LPL: linearly polarized light, CPL: circularly polarized light, LTCPC: linear-to-circular polarization conversion, R: reflection, T: transmission, A: absorption, CDR: circular dichroism of reflection, CDT: circular dichroism of transmission, EIT: electromagnetically induced transparency.

## Data Availability

The original contributions presented in this study are included in the article/Supplementary Materials. Further inquiries can be directed to the corresponding author.

## Supplementary Materials

The simulation file can be obtained from the following link: https://pan.baidu.com/s/1WQB2PpeywAOj7UZjktT9nA?pwd=0000.

## Abbreviations

The following abbreviations are used in this manuscript:

THz	terahertz
UR	unidirectional reflectionlessness
PC	polarization conversion
AT	asymmetric transmission
PSi	photosensitive silicon
PCR	polarization conversion rate
T	transmission
R	reflection
A	absorption
LPL	linearly polarized light
CPL	circularly polarized light
LTCPC	linear-to-circular polarization conversion
CDR	circular dichroism of reflection
CDT	circular dichroism of transmission
EIT	electromagnetically induced transparency
